# The case for metacognitive reflection: a theory integrative review with implications for medical education

**DOI:** 10.1007/s10459-023-10310-2

**Published:** 2024-02-12

**Authors:** Jerusalem Merkebu, Mario Veen, Shera Hosseini, Lara Varpio

**Affiliations:** 1 Uniformed Services University of the Health Sciences (USUHS), Center for Health Professions Education, Department of Medicine, Bethesda, Maryland, USA; 2 Department of General Practice, Erasmus Medical Center, Rotterdam, The Netherlands; 3 Department of Family Medicine, McMaster Education Research, Innovation, and Theory (MERIT), McMaster University, Hamilton, Ontario, Canada; 4 Perelman School of Medicine, Children’s Hospital of Philadelphia, Philadelphia, PA USA

**Keywords:** Medical education, Metacognitive reflection, Reflection, Metacognition, Theory integrative review

## Abstract

The concepts of metacognitive reflection, reflection, and metacognition are distinct but have undergone shifts in meaning as they migrated into medical education. Conceptual clarity is essential to the construction of the knowledge base of medical education and its educational interventions. We conducted a theoretical integrative review across diverse bodies of literature with the goal of understanding what metacognitive reflection is. We searched PubMed, Embase, CINAHL, PsychInfo, and Web of Science databases, including all peer-reviewed research articles and theoretical papers as well as book chapters that addressed the topic, with no limitations for date, language, or location. A total of 733 articles were identified and 87 were chosen after careful review and application of exclusion criteria. The work of conceptually and empirically delineating metacognitive reflection has begun. Contributions have been made to root metacognitive reflection in the concept of metacognition and moving beyond it to engage in cycles of reflection. Other work has underscored its affective component, transformational nature, and contextual factors. Despite this merging of threads to develop a richer conceptualization, a theory of how metacognitive reflection works is elusive. Debates address whether metacognition drives reflection or vice versa. It has also been suggested that learners evolve along on a continuum from thinking, to task-related reflection, to self-reflection, and finally to metacognitive reflection. Based on prior theory and research, as well as the findings of this review, we propose the following conceptualization: Metacognitive reflection involves heightened internal observation, awareness, monitoring, and regulation of our own knowledge, experiences, and emotions by questioning and examining cognition and emotional processes to continually refine and enhance our perspectives and decisions while thoughtfully accounting for context. We argue that metacognitive reflection brings a shift in perspective and can support valuable reconceptualization for lifelong learning.

## Introduction

Medical education is rife with concepts that have traveled into our field from medical science, psychology, humanities, and social and educational sciences (Veen et al., 2020). These *traveling concepts *(Bal, 2009) are often revised and reconstructed as they move into and across our field; the denotative meaning (i.e., the dictionary definition) and connotative value (i.e., the socially informed significance) of these concepts shift along these trajectories (Kreidler, 1998). One such concept is *metacognitive reflection.* Recent literature reviews have revealed persistent use of this concept in research addressing *reflection* or *metacognition *(Hargreaves, 2016; Jalali et al., 2015; Sandars, 2009). However, these three concepts—metacognitive reflection, reflection, and metacognition—are distinct but have undergone shifts in meaning as they migrated into medical education (Veen & Tuin, 2021).

This variability can create problems for medical educators because conceptual clarity is essential to the construction of both the field’s knowledge base and its educational interventions. For instance, the Accreditation Council for Graduate Medical Education’s (ACGME) common program requirements mandate that residents demonstrate the ability “to continuously improve patient care based on constant self-evaluation and lifelong learning.” To address this mandate, graduate medical education research often focuses on questions of training and testing residents’ abilities to engage in reflection (Winkel et al., 2017), metacognition (Mitchell et al., 2009), and metacognitive reflection (Gillon & Radford, 2012). Are these investigations addressing ACGME’s self-evaluation and lifelong learning skills from foundationally different directions? Have these terms been conflated, making it difficult to tease apart which findings relate specifically to one concept or another?

Given that reflection, metacognition, and metacognitive reflection are essential skills for lifelong learning (Rhem, 2013), we set out to understand the differences between these concepts and to construct conceptual clarity for each. There are long traditions of research into reflection and into metacognition; therefore, we first review the current state of knowledge about these concepts, highlighting how they overlap while remaining distinct. Since the concept of metacognitive reflection has more recently appeared in the literature, we conducted a theory integrative review (Battistone et al., 2023; Cornoldi et al., 2014; Kuiper & Pesut, 2004; Verplanken et al., 2007) to examine this concept. Before describing the methods used for this synthesis, we begin with an overview of the literature on reflection and metacognition followed by the intersection of metacognitive reflection. This summary describes the theoretical framework shaping our study.

### Reflection: an overview of the concept

While different descriptions are used across the literature, reflection is commonly framed as an ongoing systematic, disciplined, back-and-forth mental activity of observing, questioning, analyzing, exploring, and refining thoughts/actions for gaining clarity in understanding and achieving productive outcomes (Bright, 1996; Cole & Knowles, 2000; Dewey, 1933; Fat’hi & Behzadpour, 2011; Killion & Todnem, 1991; Nguyen et al., 2014; Osterman & Kottkamp, 2004). Inherent in reflection is an *inquiry* disposition—i.e., an openness to discovery and exploration (Larrivee & Cooper, 2006; Nguyen et al., 2014). Dewey, the pioneer philosopher and educator for this concept, described reflection as the ability to think critically and reciprocally between two opposing points while suspending judgment (Dewey, 1933). He posited that reflection is an essential part of the learning process because it allows individuals to actively engage with and make important meaning out of their experiences. According to Dewey, the processes involved in igniting reflection include experiencing perplexity/doubt and wanting to investigate (e.g., to corroborate or refute) the matter in question. Reflection involves critically analyzing and evaluating experiences to gain deeper insights and understanding. For Dewey, reflection requires work: “The building blocks of reflection comprise discipline…since these habits are not a gift of nature.” Through the work of reflective inquiry, individuals can identify the underlying assumptions and beliefs that shape their actions and decisions, and they can assess the effectiveness of their actions in achieving desired outcomes. Dewey argued that reflective thinking is not limited to academic or intellectual pursuits but can be applied to any aspect of life, including moral dilemmas. In fact, Dewey also underscored the importance of action, of not simply being locked in cycles of reflection: “Application is as much an intrinsic part of genuine reflective inquiry as is alert observation or reasoning itself. There is such a thing as too much thinking, as when action is paralyzed by the multiplicity of views suggested by a situation.” He maintained that, by engaging in reflective thinking, individuals can develop more intentional approaches that balance process and product. For Dewey, then, reflection is an active and dynamic ordered process that intricately governs our actions:

Reflection involves not simply a sequence of ideas, but a consequence a consecutive ordering in such a way that each determines the next as its proper outcome, while each in turn leans back on its predecessors. The successive portions of the reflective thought grow out of one another and support another; they do not come and go in a medley. Each phase is a step from something to something. Each term leaves a deposit which is utilized in the next term.

While Dewey was a foundational and influential scholar in this area, other conceptualizations of reflection have been offered. Reflection has also been described as higher-level thinking (Lasley, 1992), as cognitive risk-taking (Schon, 1987), and as a tool for posing thoughtful and significant questions to enhance the quality of decisions (Robinson et al., 2001). As this diversity illustrates, there is no single operational definition for reflection (Fat’hi & Behzadpour, 2011). Despite the varied definitions, there are common premises behind research into reflection. For instance, one tenet is that reflection is not about developing *certainty*, but is instead focused on *exploring and questioning* one’s own thinking. Larrivee and Cooper (2006) suggested that reflection is exploration for the purpose of understanding; this view foregrounds reflection’s orientation as being focused on curiosity. Similarly, Dewey posited that by operating in a mode of protracted inquiry, reflection enables the individual to unearth blind and opaque spots in one’s thinking, to bring to light the hidden structures of one’s thinking that lie beneath consciousness (Dewey, 1933). Curiosity can serve as a tool for exposing unconscious mental models.

Another premise underpinning reflection is that distorted perceptions must be challenged and rejected because they can negatively impact the quality of one’s decision. Dewey declared that reflection enables the individual to become aware of limitations, gaps of understanding, and partial absences that exist even as they strive to make meaning (Dewey, 1933). Shapiro and Reiff proposed that reflection supports the discovery of such weaknesses and forms the basis for considering alternative perspectives (Shapiro & Reiff, 1993).

Finally, reflection is also steeped in the fundamental assumption that all ideas are subject to questioning, and none are exempt (Cole & Knowles, 2000; Nguyen et al., 2014). This mode of thinking encourages bringing to light embedded assumptions and requires critically challenging any established beliefs. This is particularly relevant for escaping *psychic prisons* (Morgan, 2007)—i.e., favored ways of thinking that become inescapable traps. Reflection confronts entrenched mental models and tests dogmas by consistently asking questions related to each phenomenon.

With these three premises as a foundation, we can see that reflection is focused on making meaning of experiences from within the situated contexts in which the experience occurs (Boud, 1999; Dewey, 1933; Kinsella, 2010; Schon, 1987). Reflection supports the development of knowledge informed and formed by reflective practice, allowing the individual to perpetually expand to a wider range of possibilities (Larrivee & Cooper, 2006). Schon proposed that one can frame and reframe problems until insightful discoveries are generated through the continuous spiral of reflection (Schon, 1987). In this recursive process, the reflective thinker generates and tests optimal solutions and pursues action to deliberately incorporate new or enhanced understanding into action (Copeland et al., 1993).

### Metacognition: an overview of the concept

Metacognition differs from reflection. Metacognition has been part of the works of the most prominent figures in psychology (e.g., Piaget & Kamii, 1978) and, across its history, it has undergone several evolutions in its conceptualization. Flavell, an early leading researcher studying metacognition, defined it as “any knowledge or cognitive activity that takes as its object, or regulates, any aspect of any cognitive enterprise” (Flavell et al., 1993). Today, metacognition is still commonly articulated as *thinking about thinking* or *cognition about cognition* (Dimmitt & McCormick, 2012; Flavell, 1979)*.* While this simple summary is widely used, a coherent definition has yet to be widely adopted (Dimmitt & McCormick, 2012; Dinsmore et al., 2008a; Schraw, 2001; Schunk, 2008; Veenman et al., 2006; Winters et al., 2008). However, two commonalities across the literature act as foundational premises for research into metacognition.

One premise, which is aligned with Flavell’s seminal work (1979), posits that metacognition consists of both knowledge and experiences. *Metacognitive knowledge* is the individual’s acquired beliefs about his or her own and others’ cognitive orientations about people, tasks, and/or strategies; the function of metacognitive knowledge is to assess the quality of any information presented (e.g., trustworthiness, coherence). To meet the cognitive demands at hand, the individual needs to assess and manage the variations in metacognitive knowledge. *Metacognitive experiences* are the conscious cognitive or affective experiences that accompany any intellectual activity (Flavell, 1979); they interact with metacognitive knowledge to inform and shape cognitive or metacognitive goals. These metacognitive experiences can occur at any time—i.e., before, during, or after a cognitive activity—and can take many different forms—i.e., short to lengthy, simple to intricate. In sum, then, metacognition consists of both metacognitive knowledge and metacognitive experiences that co-exist and mutually inform each other.

Another commonly held proposition is that metacognition involves the planning, regulating, monitoring, and controlling of cognitive processes (Martinez, 2006). This echoes Schraw and Dennison’s argument that metacognition consists of knowledge about cognition (i.e., procedural, declarative, and conditional knowledge) as well as regulation of cognition (i.e., evaluating, monitoring, debugging, and managing information) (Schraw & Dennison, 1994).

These premises are evident in Nelson and Narens’s widely employed model of metacognition which describes the interaction between two levels of processing: object-level and meta-level (Nelson et al., 1994). Object-level monitoring involves being aware of cognitive processes, being aware of strategies used during a task, and evaluating them for effectiveness. Meta-level control refers to the strategies and actions employed to regulate and adjust cognitive processes based on the results of monitoring results. Nelson and Narens’s functional approach to metacognition provides a structure for understanding how monitoring precedes control through feedback loops. These authors point out that metacognition makes learning more effective by influencing behavior at various stages of processing.

The concept of metacognition grew primarily within the domain of cognitive sciences (Cornoldi et al., 2014). Steeped in that orientation, metacognition is the awareness of and regulation of (a) our beliefs about our own and others’ thinking processes, and (b) our cognitive and affective experiences of our own thinking processes. The concept is limited to addressing the individual’s cognitive activity and the thinking about knowledge and experiences that happens within the individual (Azevedo, 2020; Cornoldi et al., 2014; Desautel, 2009).

### The overlap between reflection and metacognition

Both reflection and metacognition address psychologically oriented phenomena that govern reflectively *thinking about thinking,* but they do so in very different ways. Reflection is grounded in the active work of meaning-making in which both individuals and groups engage (Gash, 2014; Veen & Croix, 2017). It focuses on developing knowledge through constant questioning. That knowledge is never firm because reflection encourages constant curiosity and so rejects any notion of true, unequivocal knowledge. In contrast, the thinking about thinking addressed by metacognition pays careful attention to the processes of the individual. Instead of focusing on the work of meaning-making, metacognition focuses on the awareness of and regulation of cognition. There is one cognitive reality in which the individual is engaged; via metacognition, the individual strives to develop an ever more accurate understanding of that cognition.

While this distinction is present in the literature, the programs of research that address these concepts clearly illustrate how clear definitions or distinctions have consistently failed to frame their investigations (Azevedo, 2020; Kinsella, 2010). In fact, Ford and Yore (Ford & Yore, 2012) warned that “the fuzzy borders that exist between metacognition and reflection are converging.” These fuzzy borders are further complicated because metacognitive reflection is a term that is increasingly used across these bodies of literature but is also ill defined.

Similarly, the fuzzy borders between reflection and metacognition are evident in the health professions education (HPE) literature. For instance, the overlap between the two constructs is demonstrated in this definition of regulation of cognition offered by Medina et al.: “Regulation of cognition corresponded to knowledge about the ways that students plan, implement, and monitor their learning through self-reflection” (Medina et al., 2017). Here, we see that reflection is equated with the aspects of metacognition. Recently, Linsenmeyer and Long, studying participants in undergraduate medical education, presented metacognition as a type of reflection: “When students conduct reflection, they foster a way of seeing and being, a metacognitive stance toward their own thinking, and toward the structures and forces that are shaping their professional identity” (Linsenmeyer & Long, 2023). Here, metacognition is also cultivated by reflection. Significantly, much of the HPE literature offers assertions that reflection is a metacognitive process and/or the constructs subtly blend together at the margins (Cale et al., 2023; Cloude et al., 2022; Cui et al., 2019; Cutrer et al., 2013; González & Ruiz, 2012; O’Loughlin & Griffith, 2020; Sandars, 2009). Such conflations are common in the field, suggesting that the fuzzy borders between these terms that is seen in the psychology and education literature is also evident in HPE. The lack of clarity between these terms is problematic for HPE since metacognition and reflection are regarded as holding significant value, allowing students and practitioners to critically analyze their own thought processes, to identify strategies that lead to effective learning, and to make necessary adjustments for further improvement (Asadzandi et al., 2022; Medina et al., 2017; O’Loughlin & Griffith, 2020; Pusic et al., 2022). Metacognition and reflection are also valued in HPE for supporting individuals’ ability to identify and rectify biases, misconceptions, or faulty reasoning that may be hindering optimal clinical reasoning across diverse contexts (Cloude et al., 2022; Cutrer et al., 2013; González & Ruiz, 2012; Kosior et al., 2019; Kuiper & Pesut, 2004). Clearly, the problem that plagues other disciplines is evident in the HPE literature as well: although reflection and metacognition are distinct concepts, our research often fails to delineate between them. This problem is heightened because of the emergence of research into metacognitive reflection—another separate concept that intentionally combines its constituent concepts in specific ways.

### Metacognitive reflection: a concept in the making

There is rising interest in the integration of *metacognition* and *reflection* and in the use of the term *metacognitive reflection* in the medical education literature and beyond (Graber et al., 2012; Hargreaves, 2016; Hodges, 2015; Sandars, 2009); therefore, it is important to have a clear understanding of how these three terms align and diverge. We need clear definitions of these terms and of the conceptualizations underpinning them to guide research in our field. It may be that our research will require us to revise definitions. It may be that disagreements in conceptualizations can be the source of productive knowledge development. However, if we do not begin with explicit definitions and conceptualizations, we risk building more confusion than insights. The concept of metacognitive reflection is increasingly present in research addressing reflection and metacognition, but is rarely defined and often used interchangeably with its two constitutive concepts. Given this lack of clarity, we set out to analyze the literature addressing metacognitive reflection—manuscripts that offer definitions of the term and/or that offer a theory into the concept—and to synthesize the different conceptualizations found therein.

## Method

To realize this analysis and synthesis, we engaged in a theory integrative review (TIR) (Battistone et al., 2023), a type of literature review that helps define a concept and synthesize theories when many variations exist (Torraco, 2005). TIRs are designed “to critically examine theories which address a particular phenomenon, bringing two or more theories into conversation with each other in order to reformulate, integrate, or purposefully synthesize the conceptualizations offered” (Battistone et al., 2023). As a form of knowledge synthesis developed within the constructivist tradition, TIRs build a subjectively informed aggregation of the theories (and their associated definitions) addressing a particular phenomenon. We followed the four-step process for TIRs described by Battistone et al. (2023).

### Step 1: Define the phenomenon

The phenomenon of interest was metacognitive reflection. As part of our work to define this phenomenon, we have reviewed the literature on reflection and metacognition. Our research question asked: *What is metacognitive reflection?* To address this question, we also explored several subquestions: Is metacognitive reflection the inherent overlap of reflection and metacognition? What features distinguish metacognitive reflection from its named components? How can metacognitive reflection be best conceptualized to advance the purposes of medical education?

### Step 2: Create the research team

We constructed our research team to reflect specific interests and ensure that a variety of motivations informed the research. Our team consisted of medical education scholars with a range of interests and expertise. All four members of the research team actively engage in qualitative research focused on medical education. JM’s training in educational psychology and expertise in reflection and metacognition ensured that a broad range of literature was explored to inform the synthesis and that contradictions in the literature were regularly considered to shape the team’s analysis. An expert in philosophy and interdisciplinary research, MV’s expertise guided the team’s efforts to ensure that the ontological and epistemological roots of each theory and definition included in the analysis were respected and maintained. As a senior member of the medical education community, LV focused on ensuring methodological rigor, considering implications of the findings for related bodies of research, and ensuring the ontological and epistemological consistency from the original theories through to amalgamation outcomes. SH is a social scientist and education evaluation researcher with a background in psychology and so also offered expertise in metacognition. Finally, at an earlier stage we consulted with AdlC, a medical education researcher and expert on reflection. The team’s research meetings often involved discussions about the nature and depth of the interpretations we were making about reflection, metacognition, and metacognitive reflection. We also debated how the synthesis could be relevant to and influential for the field of medical education.

### Step 3: Explore and analyze the data

To identify all pertinent literature for this review, we searched PubMed, Embase, CINAHL, PsychInfo**,** and Web of Science databases. Our search included all peer-reviewed research articles and theoretical papers published as well as book chapters that addressed metacognitive reflection. The search was not restricted by date, language, or country of publication. We did not restrict the search to HPE literature; instead, we searched across disciplines and fields of inquiry since metacognitive reflection is a relatively new concept being used in the literature. (According to our search, the term *metacognitive reflection* was first used by Karmiloff-Smith in 1979, while Dewey’s descriptions of reflection date back to the early 1930s.) Recognizing that it can take decades for conceptualizations of complex concepts like metacognitive reflection to become stable, we looked across domains in hopes of seeing how the term is stabilizing (or has stabilized) in any domain. With assistance from a university research librarian, a search of electronic databases was conducted in October 2020 and was rerun to capture new publications in September 2022. Search terms included “metacognitive reflection,” “metacognition” AND “reflection.”

This search identified 1133 papers; after deduplication, 733 articles remained in the corpus. Since the purpose of the review was to synthesize theories and definitions of metacognitive reflection, our initial review process involved determining which articles contained sufficiently rich descriptions to act as data for analysis. Articles that did not offer explicit conceptualizations or definitions of metacognitive reflection were excluded. For instance, we excluded articles that used the term *metacognitive reflection* as an adjective describing a different term (Seppanen, 2022) and papers focused solely on metacognition (Larkin, 2009; Pressley, 2005) or reflection (Grushka et al., 2005; So et al., 2018) that failed to directly or indirectly discuss metacognitive reflection. We also excluded articles addressing Metacognitive Reflection and Insight Therapy (MERIT)—a psychotherapeutic approach used with patients with mental illnesses.

With these exclusion criteria in mind, 30 articles from the corpus were sampled and reviewed by JM and AdlC, resulting in 14 conflicts. Resolving these conflicts began through conversation with MV, who read these 14 manuscripts. This high number of conflicts highlighted the high variability in the use of the term metacognitive reflection across the corpus and the implications of that variability—e.g., if we adhered too closely to one author’s definition, then many other authors’ conceptualizations (and their manuscripts) would be removed from the corpus; if we incorporated some definitions, then there was no difference between metacognitive reflection and either reflection or metacognition. LV then joined these conversations, working collaboratively with the team to analyze definitions across the 30 articles to understand core aspects of the descriptions of metacognitive reflection. Once consensus was achieved, 9 of the 30 articles were identified for full review and an approach for reviewing the titles and abstracts for inclusion markers was established. Next, two authors (JM, SH) appraised the title and abstract of all remaining papers in the corpus, excluding 636 articles that failed to meet the inclusion criteria, thereby leaving 97 articles for full-text review. During full-text review, 12 manuscripts were excluded from the corpus but, via hand searching of references and updating the search, 7 articles were added. Ultimately, 87 articles comprised the corpus for the TIR.

### Step 4: Integrate the literature

The full research team was involved in analyzing each paper in the corpus to identify the definition of metacognitive reflection presented and any underlying theory (i.e., premises that connected in a logical manner to address metacognitive reflection). In keeping with Parse’s criteria for studying theory (Parse, 2005), we focused on the structure of these theories (i.e., historical origins, foundational assumptions, principal conceptualizations, and relational statements) and their processes (i.e., the coherence, integrations, and heuristic potential of the theories). In this integration work, the research team sought to make clear how some authors’ definitions clustered, and the key aspects of those definitions that separated different clusters. This work involved looking for illustrative cases of each cluster and contrasting them with negative cases which then became the foundation for identifying a cluster that had a notably different definition. We also focused on different structures and processes of the theories offered in each of these clusters. Once these definitions, structures, and processes were made clear, we then engaged in analysis across these clusters to identify key features, principles, and premises of metacognitive reflection.

## Results

Our analysis revealed that (a) metacognitive reflection has been used to address the ways in which the concepts of reflection and metacognition overlap; and (b) researchers are increasingly interested in understanding this overlap (Alt & Raichel, 2020; Barley, 2012; Lonie & Desai, 2015a; McCabe & Olimpo, 2020; Sawicki & Wegener, 2018). This overlap consists of attention to the examination of metacognition and of internally oriented thinking—i.e., reflectively thinking about thinking. However, as illustrated in (Table 1 ; Candy et al., 1985; Davis, 2000; Dinsmore et al., 2008b; Hargis & Marotta, 2011; Sandars, 2009; Seifert, 2007; Siddiqui & Dubey, 2018), many scholars have conflated the terms *reflection* and *metacognition* in their research (i.e., they use the term *reflection* to define *metacognition* and vice versa). Further complicating this situation is that some researchers offer disparate conceptualizations, and others have even begun to informally reconceptualize reflection and metacognition as a single concept—i.e., as metacognitive reflection (Bormotova, 2010; Gillon & Radford, 2012; Hargis & Marotta, 2011). This makes clearly articulating metacognitive reflection as a distinct concept more challenging because much of the literature fails to account for the fact that reflection and metacognition are themselves different concepts. In other words, we contend that simply conceptualizing metacognitive reflection as the overlap between reflection and metacognition fails to capture its uniqueness (Granville & Dison, 2005).

**Table 1:** Conceptual definitions that explicitly link metacognition to reflection

Authors	Overlapping definitions
Davis. (2000, p. 343)	“**Reflection** as **metacognition** was the heart of the matter.”
Seifert (2007, p. 15)	“**Metacognition** and **metacognitive reflection** are, therefore, considered to be constructs that enable the learner to think about his or her thinking, and this type of **reflection** may impact learning outcomes if encouraged and developed.”
Sandars (2009, p. 685)	**“Reflection** is a **metacognitive** process that creates a greater understanding of both the self and the situation so that future actions can be informed by this understanding.”
Dinsmore et al. (2008b, p. 18)	“**Metacognition** deals primarily with **reflective** abstraction of new or existing cognitive structures.”
Hargis and Marotta (2011, p. 36)	“**Reflection** is an ideal activity to encourage **metacognitive** insight.”
Siddiqui and Dubey (2018, p. 485)	**“Metacognition** involves deep **reflection** on the cognitive processes and then regulation of those processes to maximize learning. **Metacognitive** skills help learners to **reflect** on the task at hand and also in action and on action; it also helps learners to reflect on their own **reflections**, thus leading to learning that is self-directed, goal oriented and self-evaluated.”
Candy et al., (1985, p. 141)	**“Reflection** is thus ‘meta-thinking’ (**thinking about thinking**) in which we consider the relationship between our thoughts and action in a particular context.”

### The case for metacognitive reflection as a distinct concept

Our analysis of the corpus revealed that the work of conceptually and empirically delineating metacognitive reflection as a concept separate from both reflection and metacognition has begun. Verplanken et al. are among the few researchers who have described that delineation:

Metacognitive reflection refers to the appraisal, monitoring, or control of one’s cognitions or mental functioning where various types of metacognitions may be distinguished. For instance, one may reflect on the target of thoughts, the origin of thoughts, the amount of thoughts, the valence of thoughts or consequences of thoughts (Verplanken et al., 2007).

Here, the definition for metacognitive reflection is deeply rooted in the concept of metacognition, where individuals ascend from one layer to another by adapting their cognitions (Drigas et al., 2022a).

We suggest that, in keeping with this orientation, it is useful to conceptualize metacognitive reflection as starting with processes of metacognition. Then, in metacognitive reflection, the individual moves beyond metacognition to engage in cycles of reflection—i.e., learners examine their thinking to uncover assumptions and constructs behind their actions to constantly question their strategies. These reflections add to metacognition an awareness and consideration of context, emotions, and other factors (Drigas et al., 2022b; Wynn et al., 2019).

Therefore, to conceptualize metacognitive reflection, we can use the aforementioned definition from Verplanken et al. (2007) as a starting point, which we then enhance with work from Granville and Dison, who state:

Reflection becomes metacognitive when it involves evaluating one’s own thinking processes. Metacognitive reflection goes beyond mere information processing; it concerns awareness of the thinking and the learning; it is learning to learn, evaluate, and correct the information processing. Metacognitive reflection happens when the reflection becomes more articulated, elaborated, and creative; it goes beyond the task itself to the wider implications of the work at hand (Granville & Dison, 2005).

As this excerpt illustrates, Granville and Dison bring the concepts of reflection and metacognition together in an additive way to define metacognitive reflection. They take the idea of metacognition (i.e., the monitoring, regulation, and awareness of our knowledge and experiences) and add the processes of reflection (i.e., reflecting to support learning—that is beyond knowledge—via constant elaboration).

Additionally, we can enrich this definition through Cornoldi’s et al. (1998) and Grossman’s (2009) work, thereby underscoring how metacognitive reflection also involves an affective component. Cornoldi proposed:

Metacognitive reflection is not only represented by its most evident, aware, verbalizable portion; it also includes a part not so easy to verbalize that refers to affective characteristics that include: intuitions, sensations, emotions, autobiographical memories, and self-evaluations (Cornoldi et al., 1998).

Further enhancing this appreciation for affective characteristics, Grossman (2009) described how the individual’s mental structures change when moving from metacognitive to more intensive or transformative reflection levels. Grossman described this movement as a mature psychological space that allows inner experience (i.e., thoughts, perceptions, affect, and actions) to be an object available for responsible, self-authored, higher consciousness-driven, reflective observation which has the capacity to change one’s frame of reference. Grossman emphasized that *reflecting on one’s thoughts and feelings is not a simple process of learning to make new distinctions; it requires a transformation in the way the mind is organized*.

Finally, metacognitive reflection accounts for additional reflective dimensions such as context. We can harness the work of Mason et al. (2010) and Sawicki and Wegener (2018) to again enhance the definition of metacognitive reflection to account for these other factors. Mason, Boldrin, and Ariasi considered metacognition as

a reflective activity about knowledge and knowing in the finer-grained and context sensitive spaces in which they are activated … since different contexts trigger different resources.… Metacognition in context provides some preliminary evidence that high self-reflection in learning from multiple sources may also help the activation of more sophisticated beliefs in evaluating the knowledge at hand (Mason et al., 2010).

Similarly, Sawicki and Wegener defined metacognitive reflection as pertaining to *one’s consideration of how a setting, thought, or action would affect one’s metacognitions* (Sawicki & Wegener, 2018).

With this enhanced set of considerations in mind, it appears that metacognitive reflection does involve aspects of reflection and metacognition, but that it is also distinct from those two concepts. Metacognitive reflection can take various forms and can vary greatly depending on the factors that influence the reflection activity that follows metacognition (e.g., the emotional range in the reflection activity that colors the metacognitive work).

### The missing theory of metacognitive reflection

While our integrative analysis has allowed us to merge several threads in the literature to develop a richer conceptualization of metacognitive reflection, engaging in the synthesis of insights into metacognitive reflection and theorizing how it works is a more elusive goal. Some researchers, whose arguments we have incorporated in our conceptualization, suggest that metacognition drives reflection (Grossman, 2009) and argue that “metacognitive activities … engage and encourage the development of reflection” (Lonie & Desai, 2015b). Conversely, others propose that reflection promotes metacognition and, accordingly, metacognitive capacity is developed by promoting reflection (Gonullu & Artar, 2014; Tarricone, 2011). For instance, Tarricone’s work suggests that a dialectical connection exists between metacognition and reflection but that reflection is a facilitator of metacognition (Tarricone, 2011). Adding to this confusion, some researchers focus on metacognition being a component of reflection and vice versa (Quintana et al., 2005; Siddiqui et al., 2020; Waghmare et al., 2016), while others use the terms synonymously (Alt & Raichel, 2020; Hamm, 2014; Kuiper & Pesut, 2004; Levin, 1995; Lewis, 2019) and, finally, others acknowledge that they are distinct lines of research (Barley, 2012; Bartimote-Aufflick et al., 2010; McAdoo & Manwaring, 2009; Mitchell et al., 2009; Walters et al., 2015).

These are just some of the ways reflection and metacognition are conflated. No clear premises cut across the literature to help us understand and theorize metacognitive reflection as a separate concept. In other words, sometimes the term *metacognitive reflection* is inferred to capture elements of both constructs (Cacciamani et al., 2012; Desaute, 2009; Lysaker et al., 2019; Mitchell et al., 2009; Molesworth et al., 2011; Sawicki & Wegener, 2018; Scoresby & Shelton, 2014), and other times it is mentioned but not defined, in the absence of both constructs (Makalela, 2015; Mason et al., 2010; Salovich & Rapp, 2021). There are variations in definitions; for instance, Becker et al. (2023) conceptualized metacognitive reflection as “guiding people to systematically reflect on their decision-making strategies,” whereas Moshman characterized it “as an awareness of one’s own inferences and to recognize inference as a distinct source of knowledge” (Moshman, 1991). To date, the clearest work in this space has come from Granville and Dison, who suggested that learners evolve along on a continuum from thinking, to task-related reflection, to self-reflection, and finally to metacognitive reflection (Granville & Dison, 2005). Therefore, Granville and Dison’s work (Granville & Dison, 2005) is aligned with our thinking: metacognitive reflection begins with metacognition and then is furthered by cycles of reflection that bring awareness to factors such as emotions and context.

## Discussion

We conducted this TIR to offer some conceptual gardening (Veen & Croix, 2023) by constructing a lucid conceptualization of metacognitive reflection. To answer our research question—What is metacognitive reflection?—we argue that a productive conceptualization of metacognitive reflection is one that holistically captures elements of metacognition, reflection, and the accompanying emotions involved in that work. While variability and contradiction are rife, we argue that there is a productive way forward, but it does require taking a stance to align with a subset of authors working in this area. Therefore, in keeping with the work of Wald (2015), Chick et al. (2009), Hall and Higgins (2005), Granville and Dison (2005), and Merkebu et al. (2023), we propose the following conceptualization:

Metacognitive reflection involves heightened internal observation, awareness, monitoring, and regulation of our own knowledge, experiences, and emotions by questioning and examining cognition and emotional processes to continually refine and enhance our perspectives and decisions while thoughtfully accounting for context.

We offer this conceptualization of the phenomenon to support investigations into metacognitive reflection as a distinct phenomenon. We hope that this definition can help clarify how metacognitive reflection is foundationally different from reflection and metacognition and so should not be conflated with either term. We acknowledge that we offer a limited synthesis of the definitions and theories of metacognitive reflection; however, we note that this description is limited because, unfortunately, there is much variability in the limited literature available for integration. Therefore, we suggest that the definition we offer could serve as a foundation for future inquiry that would work to build a robust theory of metacognitive reflection. Or, if future research shows that this definition does not hold up, then we offer it as a starting point for either supporting or eschewing. Whether the definition holds in the future remains to be seen, but we hope it contributes to a clearer set of future research agendas.

As Dewey remarked, “In natural growth each successive stage of activity prepares unconsciously, but thoroughly, the conditions for the manifestation of the next stage” (Dewey, 1933). We contend that engaging in metacognition is a necessary first step from which the individual can then engage in deeper levels of reflective meaning-making. Thus, metacognitive reflection is employed to characterize the monitoring and regulation aspects of metacognition and the subsequent meaning-making process of reflection, which also involves astute awareness of emotions and context. In other words, we propose that metacognitive reflection begins with the process of metacognition and then is enhanced with cycles of reflection that bring additional considerations into the process. This, therefore, is how metacognitive reflection brings a shift in perspective—i.e., metacognition is thinking about thinking which is then *augmented* with the work of reflection.

We acknowledge that our position on metacognitive reflection is most aligned with the work of many others who frame reflection as a larger and more holistic construct than metacognition and as linked to transformative learning, self-regulated learning, spiritual intelligence, faith, higher-level awareness, transcendence, moral consciousness, reflexivity, and beyond (Baumgartner, 2001; Bhaskar, 2013; Bleakley, 1999; Branson, 2007; Drigas et al., 2022a; Hetzner et al., 2011; Korthagen & Vasalos, 2009; Merkebu et al., 2023; Mezirow, 1994; Nys, 2002; Smith, 2011; Travis & Shear, 2010). Our position is most aligned with others who have posited that the work of metacognition is foundational to self-reflective monitoring because “without being able to at least describe the contents of one’s own mind, a reflection on those contents may not occur” (Demick & Andreoletti, 2012). Therefore, we propose that metacognition is the primary mechanism which then positions the individual to be able to engage in reflection. If we can imagine that metacognitive reflection involves an ascension of reflective activity, then we can conceive that researchers are desiring to capture what happens when metacognition is enhanced by the multitude of considerations that are part of the work of reflection (Merkebu et al., 2023).

### Theoretical and practical implications

Based on prior theory and research, as well as the findings of this TIR, we suggest that when we cross the thresholds from cognition to metacognition to reflection, we move into a space where we can effectively regulate our thoughts and emotions (Merkebu et al., 2023). In this space, individuals can engage in deeper levels of reflection, enabling the development of awareness and novel insight. From this standpoint, metacognitive reflection is a whole-person perspective that considers both metacognitive regulation and wider reflective perspectives. An important consequence of this proposition is that it promotes a directionality to the work of metacognitive reflection: this work starts from basic metacognition and moves to more in-depth and intensive transformational levels. This orientation recognizes the need for authentic growth and transformation (Drigas et al., 2022a; Grossman, 2009).

Given the new definition and conceptualization we offer, what are the implications for medical education? As Mieke Bal pointed out, the value of concepts is in what we do with them and how we can work with them. Metacognitive reflection can support valuable reconceptualization for lifelong learning. However, our analysis suggests that it is not appropriate for the literature to continue to conflate the terms metacognition, reflection, and metacognitive reflection. They are distinct concepts. Further, given their distinctions, our research findings suggest that medical educators should teach learners to engage in these processes at different times. For instance, perhaps it is most reasonable, at the undergraduate medical education level, to ask students to engage in metacognition. We propose that metacognition is the first step in learning how to question the knowledge we hold; it requires the learner to be aware of the ways in which we are thinking and how we are using our thinking to productively engage in learning. Then, students move through the medical education continuum and develop more advanced knowledge, skills, and attitudes, including adding reflection to their metacognition work. Perhaps when learners are at the end of their undergraduate training or entering graduate training, they can be expected to develop the ability to sustain metacognitive development and ascend from one layer to another via the complexities and nuances involved in reflection (Drigas et al., 2022a). In this way, medical learners progress towards an end goal of being capable of engaging in metacognitive reflection: first they develop robust metacognitive skills and then they develop rich reflection skills that they harness to enhance their metacognition. In this way, the journey to becoming lifelong learners follows the journey of mastering metacognitive reflection skills.

This conceptualization of metacognitive reflection also offers medical educators the opportunity to identify why some medical learners might struggle to develop this important skill and how to engage in remediation efforts. Do learners struggle with foundational metacognition skills? If so, they might be guided to consider the differences between their metacognitive knowledge and metacognitive experiences. The educator can then help them develop skills to plan, regulate, monitor, and control their cognitive processes. However, if learners have sound metacognition skills, remediation can focus on their ability to engage in high-quality or productive reflection (El-Dib, 2007). Remediation effort would focus on helping learners develop the ability to make meaning of experiences from within situated contexts, continually expanding to consider a wider range of possibilities through spirals of reflection.

Finally, the role of emotion is different in metacognition, reflection, and metacognitive reflection. As argued by Shapiro, much of medical education’s hidden curriculum has encouraged students “to separate and distance themselves” from their own emotions and those of others. However, as the analysis in this paper suggests, if we understand when emotions come into play in metacognitive reflection—and indeed, in both metacognition and reflection—we can thoughtfully bring emotions back into each individual’s consideration at appropriate times. For instance, we contend that metacognition is a first step for engaging in reflective meaning-making. Therefore, metacognitive awareness of emotions is part of the very initial workings of these processes. Instead of divorcing emotions from this work, we argue that emotions are part of the primary processes, which individuals need to productively reappraise and regulate in order to embark on reflective meaning-making.

### Limitations and future directions

This review addresses a gap in the metacognitive reflection literature; however, as our synthesis efforts revealed, the theory of metacognitive reflection and the processes by which it works have not been the focus of much research attention. The lack of literature in this area poses a limitation in our review. Important questions remain to be addressed: How do metacognitive processes carry over to impact engagement in reflection? Ford and Yore (2012) have cautioned that these constructs are converging “as the move toward constructivism has necessitated critical considerations of knowledge about thinking.” Should we conceptualize metacognitive reflection from an objectivist, subjectivist, or pragmatist epistemology? What would be the implications of choosing one orientation over another? Future research could address these gaps by studying what constitutes metacognitive reflection to develop robust theory. Furthermore, if the conceptualization of metacognitive reflection as we have described it is embraced by the community, research will be needed to construct instruments that measure individuals’ competency in this area, since it is a foundational competency required of practicing physicians.

### Conclusion

This TIR offers a synthesis of the literature addressing metacognitive reflection. We offer a new definition of metacognitive reflection and highlight its salient features. We suggest a conceptualization that places metacognition as the first and foundational aspect of metacognitive reflection, from which individuals can then engage in iterative cycles of reflection. Our knowledge synthesis provides a coherent conceptualization of metacognitive reflection and proposes how this construct can be leveraged to serve medical education. Additionally, this review highlights that metacognition and reflection are not synonyms. They are related but distinct constructs that should not be used interchangeably.
